# Heat Acclimation Does Not Modify *Q*_10_ and Thermal Cardiac Reactivity

**DOI:** 10.3389/fphys.2019.01524

**Published:** 2019-12-17

**Authors:** Bernhard Kampmann, Peter Bröde

**Affiliations:** ^1^Department of Occupational Health Science, School of Mechanical Engineering and Safety Engineering, University of Wuppertal, Wuppertal, Germany; ^2^Department of Immunology, Leibniz Research Centre for Working Environment and Human Factors (IfADo), Dortmund, Germany

**Keywords:** heat acclimation, metabolic rate, heart rate, body temperature, rectal temperature, *Q*_10_ coefficient, heat strain, heat stress

## Abstract

Heat acclimation (HA) is an essential modifier of physiological strain when working or exercising in the heat. It is unknown whether HA influences the increase of energy expenditure (*Q*_10_ effect) or heart rate (thermal cardiac reactivity TCR) due to increased body temperature. Therefore, we studied these effects using a heat strain database of climatic chamber experiments performed by five semi-nude young males in either non-acclimated or acclimated state. Measured oxygen consumption rate (VO_2_), heart rate (HR), and rectal temperature (*T*_re_) averaged over the third hour of exposure were obtained from 273 trials in total. While workload (walking 4 km/h on level) was constant, heat stress conditions varied widely with air temperature 25–55°C, vapor pressure 0.5–5.3 kPa, and air velocity 0.3–2 m/s. HA was induced by repeated heat exposures over a minimum of 3 weeks. Non-acclimated experiments took place in wintertime with a maximum of two exposures per week. The influence of *T*_re_ and HA on VO_2_ and HR was analyzed separately with mixed model ANCOVA. Rising *T*_re_ significantly (*p* < 0.01) increased both VO_2_ (by about 7% per degree increase of *T*_re_) and HR (by 39–41 bpm per degree *T*_re_); neither slope nor intercept depended significantly on HA (*p* > 0.4). The effects of *T*_re_ in this study agree with former outcomes for VO_2_ (7%/°C increase corresponding to *Q*_10_ = 2) and for HR (TCR of 33 bpm/°C in ISO 9886). Our results indicate that both relations are independent of HA with implications for heat stress assessment at workplaces and for modeling heat balance.

## Introduction

Heat acclimation (HA) refers to adaptations of physiological functions to repeated exposures to heat stress enhancing the tolerance to that stressor and, thus, reducing physiological strain (Taylor, 2014). This manifests, among others, in increased sweat rates accompanied by reduced rates of energy expenditure, heart rates, and body temperatures when exercising in the heat with relevance in military (Sawka et al., 2011), occupational (Strydom et al., 1966; Kampmann, 2000), or sports context (Garrett et al., 2011; Périard et al., 2015).

The above-mentioned heat strain indicators are interlinked, e.g., by the well-known temperature dependency of the rates of chemical and physiological processes (van’t Hoff, 1884), which is conveniently described as *Q*_10_ coefficient, defined as “*the ratio of the rate of a physiological process at a particular temperature to the rate at a temperature 10°C lower*” (IUPS Thermal Commission, 2003). Using oxygen uptake rate (VO_2_) as indicator of metabolic rate and rectal temperature (*T*_re_) characterizing body temperature, this is mathematically expressed as (Chaui-Berlinck et al., 2002):

(1) $$Q_{10}={\left(VO_{2}/VO_{2,ref}\right)}^{10/\left(T_{re}-T_{re,ref}\right)}$$

VO_2,ref_ refers to the oxygen uptake rate at a reference rectal temperature, e.g., *T*_re,ref_ = 36.8°C. Re-arranging Eq. 1, it expresses percentage change in oxygen uptake rate (%VO_2_) due to a change in *T*_re_ (*ΔT*_re_) as follows (Bröde and Kampmann, 2019):

(2) $$\%{VO}_{2}=\left({Q_{10}}^{△T_{re}/10}-1\right)\times 100$$

*Q*_10_ coefficients for biological systems typically vary between 2 and 3 (Chaui-Berlinck et al., 2002; IUPS Thermal Commission, 2003; Seebacher et al., 2015), with relevance not only during hyperthermia (Nadel et al., 1971; Howells et al., 2013), but also during body cooling (Erecinska et al., 2003). Furthermore, the setting *Q*_10_ = 2 is applied in human thermoregulation models (Werner and Buse, 1988; Fiala et al., 2012). A recent study on the influence of core temperature on oxygen uptake with 11 young acclimated males (Kampmann and Bröde, 2015) confirmed this with *Q*_10_ = 2.1 on average, corresponding to a 7% increase in VO_2_ per degree rise in *T*_re_ according to Eq. 2. However, there was large inter-individual variation from *Q*_10_ = 1, i.e., no increase in VO_2_ due to *T*_re_, to *Q*_10_ = 8, corresponding to 23% VO_2_ increase per degree rise in *T*_re_.

There are reports on decreased *Q*_10_ after acclimation to heat indicating a reduced sensitivity of metabolic rates to increasing environmental temperature in ectotherms (Sandblom et al., 2014; Seebacher et al., 2015). Aiming at a comparative human study related to body temperature, we would like to extend the preceding works and analyze “whole organism” *Q*_10_ effects with acclimated compared to non-acclimated participants.

Body temperature also influences heart rate (HR) with a typical increase of 30–40 bpm per degree rise in *T*_re_ (Vogt et al., 1973; Kuhlemeier and Miller, 1978; Kampmann, 2000; ISO 9886, 2004; Bröde and Kampmann, 2019). This increase is termed “thermal cardiac reactivity” (TCR), and also “thermal pulses” (Kampmann et al., 2001) or “thermal heart rate component” (ISO 8996, 2004; Dubé et al., 2019), and shows considerable inter-individual variation between 16 and 60 bpm/°C (Kampmann, 2000; Bröde and Kampmann, 2019). An earlier study (Kuhlemeier and Miller, 1978) estimated TCR from pooled intra- and inter-individual data under different workloads of workers classified in “hot” and “cold-neutral” professions during summer and winter months, thus considering “natural” acclimation effects. The authors reported 6–7 bpm lower HR in summer compared to winter, and a 5–6 bpm reduction in HR in “hot” professions compared to the reference group, but did not allow for changes in the slope, i.e., TCR, depending on acclimation in their analyses, which were performed using the estimated overall value of 29 bpm/°C. Thus, it is unclear, whether acclimation changes TCR.

*Q*_10_ and TCR are relevant for the assessment of thermal stress and strain in different fields of application, e.g., as a potential source of error when estimating metabolic rate from heart rate measurements (ISO 8996, 2004; Malchaire et al., 2017). Here, TCR may induce an overestimation bias (Bröde and Kampmann, 2019) requiring dedicated correction procedures (Vogt et al., 1973; Kampmann et al., 2001; Dubé et al., 2019). *Q*_10_ also helps to explain the reduced cycling gross efficiency observed with increasing body temperature (Daanen et al., 2006).

Recently, *Q*_10_ and TCR were explicitly and implicitly applied for the non-invasive determination of core temperature from peripheral signals including heart rate, sometimes also involving the estimation of metabolic rate. Algorithms have been developed typically for work in protective clothing in industry (Richmond et al., 2015), firefighting (Kim, 2018), and military scenarios (Buller et al., 2013; Welles et al., 2018; Hunt et al., 2019).

For those applications, it is important to know whether the underlying algorithms will require adjustments considering the heat acclimation state of the individual. A recent pooled analysis (Bröde et al., 2009) of the changes in *T*_re_ (*ΔT*_re_) and HR (ΔHR) after 5 days of short-term HA observed in 23 females and 34 males showed a significant positive correlation with ΔHR increasing with Δ*T*_re_ by 32.6 bpm/°C, close to the 33 bpm/°C reported for TCR in international standards (ISO 9886, 2004). Thus, TCR may have a role in explaining the effects of HA. However, it is unknown whether *Q*_10_ or TCR will depend on HA.

Therefore, the aim of this research was to study the influence of HA on *Q*_10_ and TCR using an extensive heat strain database compiled from controlled climatic chamber experiments.

## Materials and Methods

### Heat Strain Database

**Figure 1:**
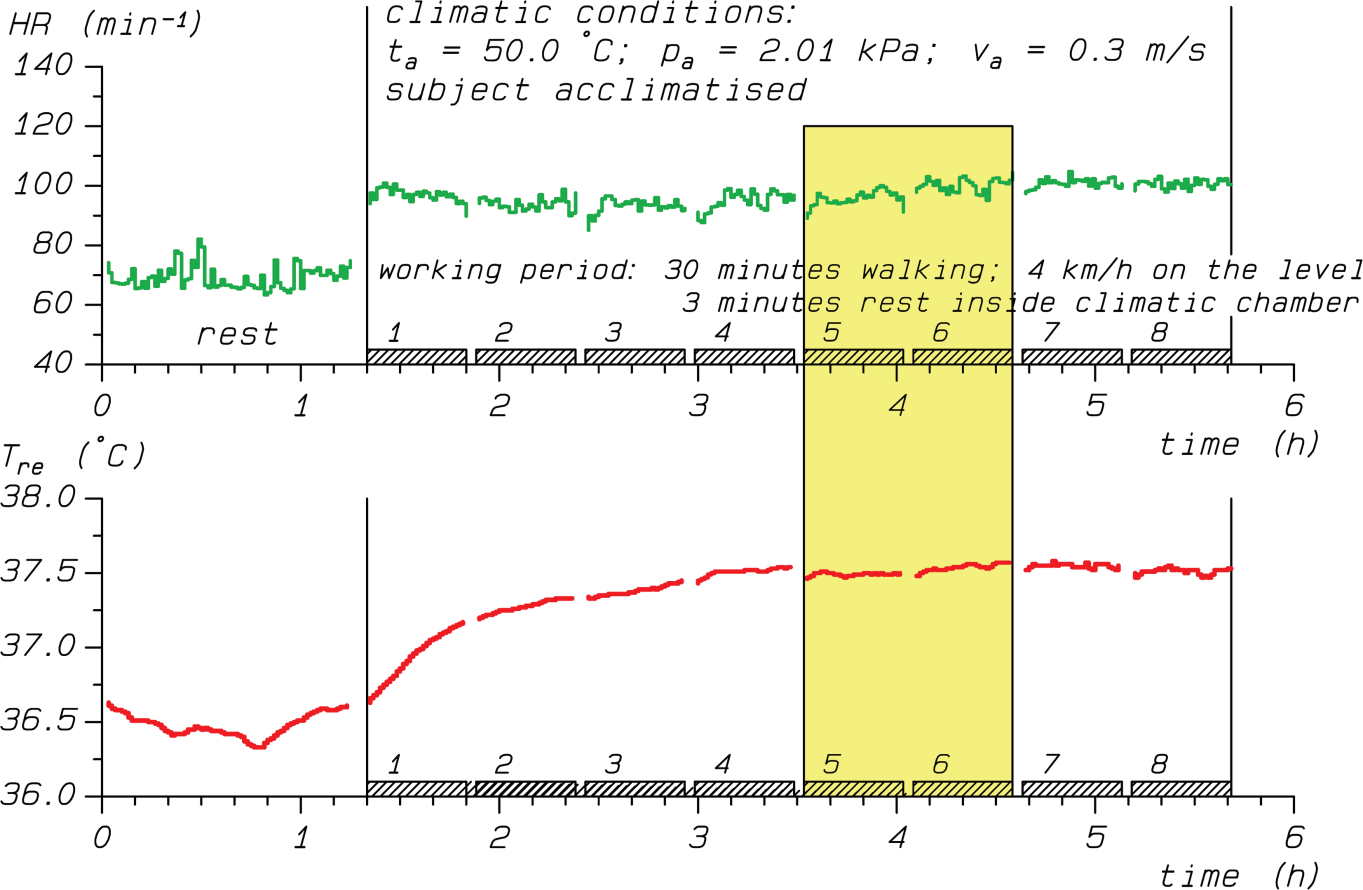
Time course of heart rate (HR, upper panel) and rectal temperature (*T*_re_, lower panel) during a heat stress experiment. The yellow shaded area marks the time interval with averaged values of HR = 98 bpm and *T*_re_ = 37.5°C used for analyses, while VO_2_ (not shown) was 714 mL/min.

We used a heat strain database of climate chamber experiments conducted previously at IfADo (Wenzel et al., 1989; Kampmann, 2000) according to the ethical principles of the Declaration of Helsinki with approval by IfADo’s local Ethics Committee. Figure 1 illustrates the recordings of rectal temperature (*T*_re_) and heart rate (HR) for a typical heat stress exposure.

We searched our database for individuals having performed series of experiments in both non-acclimated (HA0) and acclimated (HA1) states. Inclusion criteria were a minimum number of 15 experiments per series with comparable workload and clothing in order to determine *Q*_10_ and TCR on an individual level. We retrieved 273 trials organized in 10 series, which originated from five semi-nude young fit males in either HA0 or HA1 state. The number of experiments in each series varied depending on acclimation state and individual between 15 and 47 experiments, with total figures of 118 trials for HA0 and 155 for HA1. The personal characteristics (mean ± SD) of the participants were 20.2 ± 0.8 years of age, 1.84 ± 0.02 m of body height, 71.4 ± 7.5 kg of body weight, 1.9 ± 0.1 m^2^ of body surface area, and 47.1 ± 9.8 mL/min/kg of peak rate of oxygen uptake.

As the procedures have been described in detail elsewhere (Kampmann, 2000), they are only briefly summarized here. Each trial consisted of treadmill work with constant workload of walking 4 km/h on the level for at least 3 h organized in 30 min work periods interrupted by 3 min breaks for determining body weight loss (Figure 1). The participants were exposed to varying levels of heat stress with conditions characterized by different combinations of air temperature (range 25–55°C), water vapor pressure (0.5–5.3 kPa), and air velocity (0.3–2.0 m/s). Mean radiant temperature was equal to air temperature.

Rectal temperatures were recorded continuously using a thermistor probe (YSI 401, Yellow Springs) inserted 10 cm past the anal sphincter, as well as heart rates, which were obtained using ECG electrodes. *T*_re_ and HR were stored as 1-min averages, and means calculated over the third hour of exposure were used for further analyses (Figure 1). They were matched to oxygen uptake rates (VO_2_) obtained toward the end of the third hour of exposure by collecting the expired air with Douglas bags (Douglas, 1911). We determined the oxygen and carbon dioxide concentrations with a paramagnetic gas analyzer (Servomex) and infrared analyzer (UNOR Mark 2), respectively. The VO_2_ calculations based on the Haldane transformation (Poole and Whipp, 1988) are detailed in Rutenfranz and Wenzel (1980), while the methods were historically reviewed recently (Shephard, 2017).

### Heat Acclimation Protocol

HA was induced by repeated experiments in warm-humid climates (air temperature 38–40°C with 65–70% relative humidity) over 3–4 weeks in a way that the subjects could sustain 3 h of heat exposure reaching a *T*_re_ of 38.5°C. To counteract a decay in acclimation over the weekend (Daanen et al., 2018), HA was re-established on Mondays and measurements for the series started the day after.

Non-acclimated exposures took place in wintertime in order to avoid seasonal adaptation, and with a maximum of two exposures per week on non-consecutive days to prevent short-term HA effects.

### Data Analysis and Statistics

Statistical analysis was performed using R version 3.6.1 (R Core Team, 2019). The influence of *T*_re_, which was centered to a reference value of 36.8°C, and HA on VO_2_ and HR was analyzed separately with linear mixed model ANCOVA (Bates et al., 2015). The models included random intercepts and *T*_re_ slopes for individuals nested within acclimation status with tests for statistical significance carried out applying Kenward-Roger approximations for denominator degrees of freedom (Kuznetsova et al., 2017).

## Results

**Figure 2:**
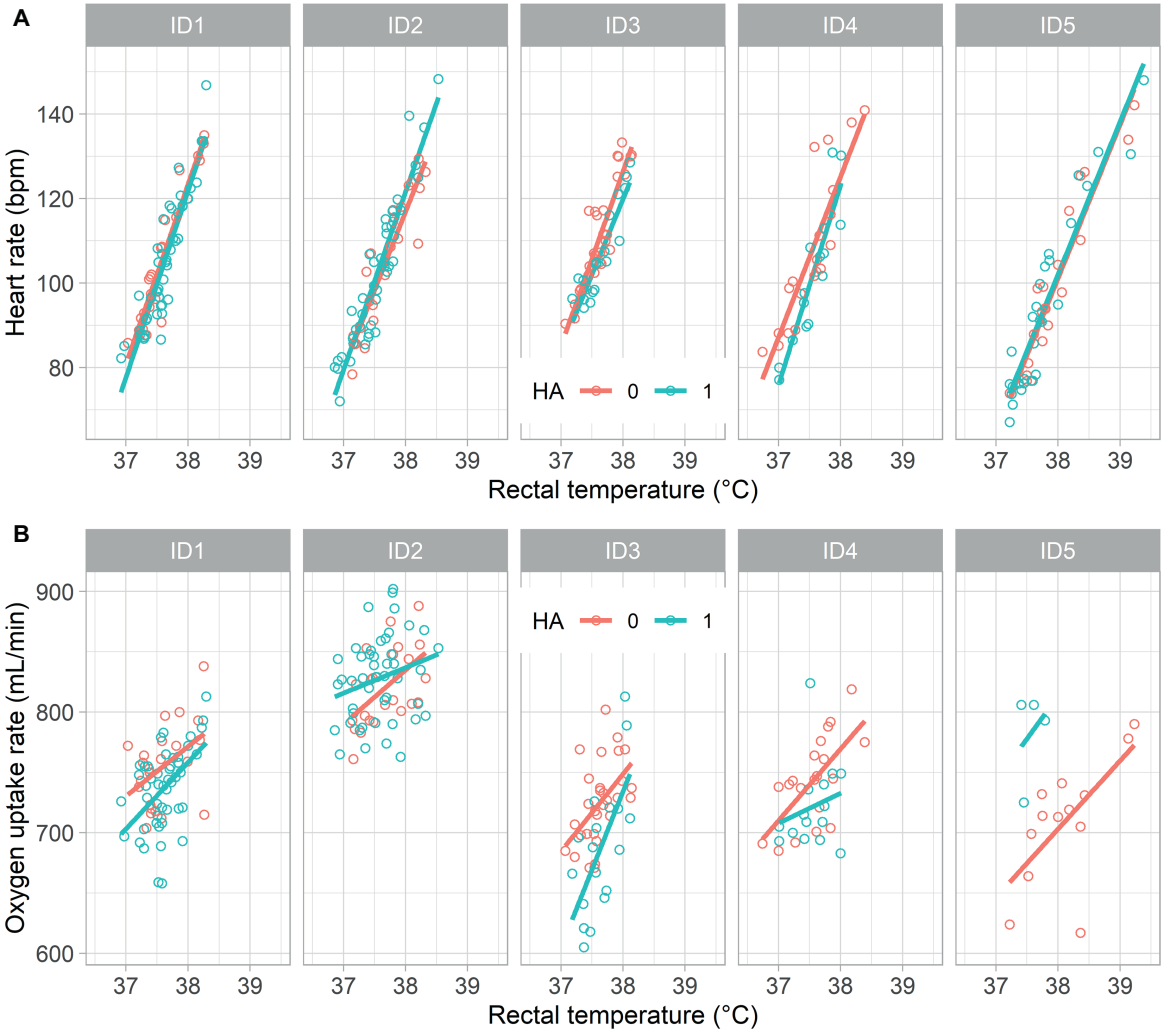
Measured values and linear regression lines illustrating the influence of rectal temperature on **(A)** heart rate (thermal cardiac reactivity) and on **(B)** oxygen uptake rate (*Q*_10_ effect) for five participants (ID1–ID5) in non-acclimated (HA0) and acclimated (HA1) states, respectively.

Figure 2 illustrates the influence of *T*_re_ on HR (Figure 2A) and VO_2_ (Figure 2B), respectively. While the information for *T*_re_ and HR was almost complete (only 5 missing HR values), 34 VO_2_ observations were missing, the majority (24) for ID5 in HA1 due to a defect in the O_2_ analyzer requiring repairing while the series of exposures had to be continued. Nevertheless, linear regression lines showed positive correlations with *T*_re_ for both dependent variables in each series.

The parameter estimates from the statistical analysis (Table 1) indicate that on average HR rose from 72 bpm at reference *T*_re_ = 36.8°C by 39 bpm per degree increase in *T*_re_, i.e. TCR was 39 bpm/°C for non-acclimated individuals. When acclimated, the intercept was reduced by 4 bpm, while TCR slightly increased to 41 bpm/°C. However, while the TCR effect was highly statistically significant (*p* < 0.0001), adjustments due to HA to both intercept and slope were non-significant (*p* > 0.4).

**Table 1:** Mixed effects ANCOVA results for the influence of *T*_re_, centered to a reference value of 36.8°C, and heat acclimation (HA) on HR (thermal cardiac reactivity) and on VO_2_ (*Q*_10_ effect).

Parameter	HR (bpm)	VO_2_ (mL/min)
Intercept @*T*_re_ = 36.8°C for non-acclimated	71.9 ± 3.3 (***p* < 0.0001**)	702.8 ± 30.5 (***p* < 0.0001**)
*T*_re_ slope for non-acclimated	39.0 ± 1.9 (***p* < 0.0001**)	50.7 ± 10.9 (***p* = 0.0024**)
HA1: intercept adjustment for acclimated	−3.6 ± 4.6 (*p* = 0.4560)	8.7 ± 43.4 (*p* = 0.8456)
*T*_re_*HA1: slope adjustment for acclimated	2.0 ± 2.7 (*p* = 0.4716)	−1.0 ± 16.5 (*p* = 0.9516)

*T_re,_ rectal temperature; HR, heart rate; VO_2_, oxygen uptake rate; HA1, acclimated. Data are parameter estimates ± SE with values of *p* for the null hypotheses of zero estimates in brackets resulting from linear mixed model analyses including random intercepts and slopes for participants nested within acclimation status. Bold p-values indicate statistically significant results (*p* < 0.05)*.

Similar to TCR, rising *T*_re_ also significantly (*p* < 0.01) increased VO_2_ by about 7% per degree increase of *T*_re_ compared to the reference VO_2_ at *T*_re_ = 36.8°C for both HA0 and HA1; neither slope (i.e., *Q_10_*) nor intercept depended significantly on HA (*p* > 0.8).

## Discussion

Our results regarding the impact of *T*_re_ on HR conform with reports of TCR between 30 and 40 bpm/°C in previous studies (Vogt et al., 1973; Kuhlemeier and Miller, 1978; Bröde and Kampmann, 2019) and in ISO 9886 (2004). They also agree with former effect sizes for VO_2_, as the observed increase of 7%/°C corresponds to a *Q*_10_ coefficient around 2, which were reported as mean value in human trials (Kampmann and Bröde, 2015) and used in advanced models of human thermoregulation (Werner and Buse, 1988; Fiala et al., 2012).

A novel finding of our study was that heat acclimation did neither modify thermal cardiac reactivity nor influence *Q_10_*.

In contrast, a *Q*_10_ decrease after acclimation to warm conditions was reported for ectotherms and interpreted as lowered sensitivity to increasing environmental temperatures under climate change scenarios (Sandblom et al., 2014). However, those lowered *Q*_10_ were calculated across states of acclimation presuming that acclimation will shift the otherwise unchanged temperature-response function to the right (Seebacher et al., 2015). The latter would conform to the invariance regarding heat acclimation of the intra-individually determined *Q*_10_ in our study. On the other hand, the shift of the intercept observed in Table 1 was minimal and non-significant.

There are limitations within this study that only used observations retrieved from an existing database of semi-nude fit young males performing light to moderate work. It would be worthwhile to verify our results involving other populations, e.g., females or elderly, under higher activity levels or working in protective clothing. Future studies might further include heart rate variability (HRV) measurements quantifying the sympathetic and vagal impacts on HR. Earlier studies had indicated vagal dominance following HA (Flouris et al., 2014), and negative correlations of vagal tone with *T*_re_ and HR (Brenner et al., 1997). However, as HRV calculations require beat-to-beat (RR) intervals, we could not perform these analyses with our aggregated HR data.

Nevertheless, our analyses of 273 experiments indicate that intra-individually determined *Q*_10_ and TCR remain unaltered following heat acclimation. This stability could have implications for the development and application of methods using the *Q*_10_ and TCR relationships for the heat stress assessment at workplaces (Malchaire et al., 2017; Bröde and Kampmann, 2019), and for the modeling of heat balance, e.g., for predicting core temperature from non-invasive signals when working with protective clothing in industrial, military, or firefighting operations (Richmond et al., 2015; Kim, 2018; Welles et al., 2018).

## Data Availability Statement

The raw data including the R scripts used for the analyses supporting the conclusions of this article are provided as Supplementary Material to this article.

## Ethics Statement

The studies involving human participants were reviewed and approved by IfADo’s Local Ethics Commission. The patients/participants provided their written informed consent to participate in this study.

## Author Contributions

BK and PB designed and conceived the analyses. BK collected the data. PB organized the database and performed the statistical analysis. Both authors interpreted the data, and wrote the manuscript, and, after critically reviewing and providing significant editing of its content, approved the final article.

### Conflict of Interest

The authors declare that the research was conducted in the absence of any commercial or financial relationships that could be construed as a potential conflict of interest.
